# Neutropenia in Childhood—A Narrative Review and Practical Diagnostic Approach

**DOI:** 10.3390/hematolrep16020038

**Published:** 2024-06-16

**Authors:** Georgios Katsaras, Silouani Koutsi, Evdokia Psaroulaki, Dimitra Gouni, Pelagia Tsitsani

**Affiliations:** 1 Paediatric Department, General Hospital of Pella—Hospital Unit of Edessa, 58200 Edessa, Greece; siliakou1@gmail.com (S.K.); evdokiapsaroulaki@gmail.com (E.P.); dimigouni@gmail.com (D.G.); pelazina@gmail.com (P.T.); 2 Paediatric Outpatient Department, Health Care Center of Aridaia, 58400 Aridaia, Greece

**Keywords:** neutropenia, neutrophils, childhood

## Abstract

Neutropenia refers to a decrease in the absolute neutrophil count according to age and race norms and poses a common concern in pediatric practice. Neutrophils serve as host defenders and act crucially in acute inflammation procedures. In this narrative review, we systematically present causes of neutropenia in childhood, mainly adopting the pathophysiological classification of Frater, thereby studying (1) neutropenia with reduced bone marrow reserve, (2) secondary neutropenia with reduced bone marrow reserve, and (3) neutropenia with normal bone marrow reserve. Different conditions in each category are thoroughly discussed and practically approached from the clinician’s point of view. Secondary mild to moderate neutropenia is usually benign due to childhood viral infections and is expected to resolve in 2–4 weeks. Bacterial and fungal agents are also associated with transient neutropenia, although fever with severe neutropenia constitutes a medical emergency. Drug-induced and immune neutropenias should be suspected following a careful history and a detailed clinical examination. Cytotoxic chemotherapies treating malignancies are responsible for severe neutropenia and neutropenic shock. Rare genetic neutropenias usually manifest with major infections early in life. Our review of taxonomies clinical findings and associates them to specific neutropenia disorders. We consequently propose a practical diagnostic algorithm for managing neutropenic children.

## 1. Introduction

Neutrophils, also known as polymorphonuclear leukocytes, are produced in the stem cells of the bone marrow [1]. About 1000–1500 × 10^6^/L neutrophils are produced daily, while their average lifespan is 7–10 days. Only 2–5% of the produced neutrophils enter the circulation, while the rest remain in the storage pool of the bone marrow [2]. They play a major role in acute inflammation and host defense against microbial pathogens [1].

In order for neutrophils’ compensatory function to be fulfilled, an adequate number of them needs to be produced in the bone marrow, while an adequate number also needs to be transferred in circulation and migrate to the area of infection [3].

Many methods have been developed regarding neutrophils’ calculation in peripheral blood that are well correlated with the gold standard method, which is their count in a peripheral blood smear: the Abbott method, where multiangle polarized scatter separation and three-color fluorescence detection are used; the Siemens method, where peroxidase staining, light scatter, and absorption are used; the Beckman Coulter method, where impedance volume/conductivity and five-angle light scatter are used; and the Sysmex method, where fluorescent staining, forward/side scatter, and side fluorescent light detection are used [4].

Neutropenia is defined as an absolute number of neutrophils less than 2500 × 10^6^/L in neonates and infants and less than 1500 × 10^6^/L in toddlers and older children and adults. Regarding African American children, this limit ranges from 1000 to 1500 × 10^6^/L [5,6].

The severity and frequency of infections are inversely correlated to the absolute neutrophil counts and directly to the prolongation in time of neutropenia. On the other hand, the risk of infection is higher when the decreased number of neutrophils is caused by a decrease in cell production in the myeloid series in the bone marrow in comparison to the decreased numbers of neutrophils due to their destruction in the peripheral blood [7].

According to the absolute neutrophil count, neutropenia is classified as mild in values 1000–1500 × 10^6^/L, moderate in values 500–999 × 10^6^/L, and severe in values <500 × 10^6^/L. There have been proposed classifications according to the benign nature of the neutropenia, the acuteness or chronicity, the age of onset, and the nature of the cause [1,4].

In the present study, we decided to follow the classification of Frater J. [4], which constitutes a classification system that takes into account the physiology of the granulocyte maturation in the bone marrow along with the course of the differentiated neutrophils in the peripheral blood and the other end organs. This system classifies neutropenia as (1) neutropenia with reduced bone marrow reserve, (2) secondary neutropenia with reduced bone marrow reserve, and (3) neutropenia with normal bone marrow reserve.

The aim of our study, apart from a narrative review regarding the different conditions/disorders that can cause neutropenia in childhood, was to provide a practical diagnostic and therapeutic approach concerning neutropenia in this sensitive age group.

## 2. Neutropenia with Reduced Bone Marrow Reserve

### 2.1. Cyclic Neutropenia

Cyclic neutropenia, or cyclic agranulocytosis, is a rare hematological disorder (1:1,000,000 in the general population) with an autosomal-dominant pattern of transmission, in which mutations occur in the gene for neutrophil elastase (ELA2 or ELANE) [8]. The disease presents with recurrent fever, deep and painful mouth ulcers, painful lymphadenopathy, and cellulitis from minor cuts on the hands and perineal areas, while sinusitis, otitis, pharyngitis, and bronchitis may often be present. Patients with cyclic neutropenia may also present with acute peritonitis (abdominal guarding, ileus, and septic shock). Between the periods of recurrent fever, mouth ulcers, and infections, patients present no pathological findings in physical examination. Typical cases of cyclic neutropenia have oscillations of neutrophils and monocytes with 21-day periodicity. During the neutropenic period, blood neutrophil levels fall to less than 200 × 10^6^/L for 3–5 days. The neutrophil count then usually increases to near the lower limit of normal, about 2000 × 10^6^/L, and remains at approximately this level until the next neutropenic period [9]. The availability of recombinant human granulocyte colony-stimulating factor (G-CSF) has greatly changed the management of cyclic neutropenia. Clinical trials clearly have established that G-CSF treatment (2–5 μg/kg/d) increases the neutrophil oscillations’ amplitude, shortens the neutropenia duration, and changes the cycle length from 21 to about 14 days, while patients have reported a reduction in recurrent fevers, mouth ulcers, and all other disease manifestations [10,11].

### 2.2. Shwachman–Diamond Syndrome

Shwachman–Diamond syndrome (SDS) is a rare autosomal recessive congenital disorder with an incidence of one in 77,000 individuals [12]. The SDS gene (7q11) mutations have been detected in 80% of patients with SDS, suggesting a heterogeneous model of transmission. The disease is characterized by pancreatic insufficiency, bone marrow dysfunction, and skeletal abnormalities. Even though no specific biochemical or genetic test is available at the moment for a definite diagnosis, evidence of exocrine pancreatic dysfunction and hematological abnormalities are the main characteristic findings. Short stature, skeletal abnormalities, hepatomegaly, or biochemical abnormalities of the liver are supportive findings of the diagnosis [13]. The clinical diagnostic criteria used by Dror and Freedman [14] are the following. (1) Exocrine pancreatic dysfunction (at least one of the following): (a) abnormal quantitative pancreatic stimulation test, (b) serum cationic trypsinogen below the normal range, and (c) abnormal 72 h fecal fat analysis plus evidence of pancreatic lipomatosis by ultrasonographic examination or computed tomography (CT) scan. (2) Hematological abnormalities (at least one of the following): (a) chronic (on two occasions at least 6 weeks apart) single lineage or multilineage cytopenia with bone marrow findings consistent with a productive defect ((i) neutrophils <1500 × 10^6^/L, (ii) hemoglobin concentration <2 standard deviations below mean, adjusted for age, and (iii) thrombocytopenia <150,000 × 10^6^/L) and (b) myelodysplastic syndrome. Management of children with SDS requires pancreatic enzymes for a significant proportion of patients. The dosage should be adapted to the severity of the symptoms, such as steatorrhea, abdominal pain, and growth parameters. Depending on the evolution of hematological abnormalities, a full blood count must be performed every 3–6 months or more frequently if symptoms require so. An annual bone marrow biopsy must be performed for surveillance of the acquisition of cytogenetic abnormalities [13].

### 2.3. Kostmann Syndrome

Severe congenital neutropenia (SCN), known as Kostmann syndrome, is a rare heterogeneous group of diseases (3–8.5 per million individuals) characterized by arrested neutrophil maturation in the bone marrow [15]. It is caused by HAX1 gene mutation, an autosomal recessive condition that displays recurrent respiratory tract, skin, and deep tissue infections from the first few months of life [16]. The arrested neutrophil maturation at the promyelocyte stage, along with severe neutropenia (<500 × 10^6^/L) and death due to bacterial infections, pose the main characteristics of the syndrome [17]. The only curative therapy is hematopoietic stem cell transplantation (HSCT), but due to the complications of this procedure, administration of G-CSF is preferable in most cases, with survival >80% of treated cases [18].

### 2.4. Chédiak–Higashi Syndrome

Chédiak–Higashi syndrome (CHS) is an inherited condition that follows an autosomal recessive pattern. Less than 500 cases have been described worldwide [19]. It is characterized by various symptoms, including frequent bruising, nosebleeds, bleeding from the gums or other mucosal surfaces, albinism affecting the skin and eyes, and recurring bacterial infections. The syndrome is caused by a mutation in a gene called lysosomal trafficking regulator protein (LYST), which results in a reduced ability to engulf and eliminate foreign particles, increasing the likelihood of recurrent bacterial infections. In the accelerated phase of the disease, fever, hepatosplenomegaly, lymphadenopathy, neutropenia, anemia, and sometimes thrombocytopenia are present. Long-term progression of the disease can lead to neurologic manifestations, such as stroke, coma, ataxia, tremor, motor and sensory neuropathies, and absent deep tendon reflexes. Most patients (90%) die within the first 10 years of life, during the accelerated phase, and due to recurrent infections. Abnormally large intracytoplasmic granules, which can be found especially in white blood cells and bone marrow, are diagnostic for the disorder. Molecular genetic testing can also be employed to identify the presence of two variants in the LYST gene, which is associated with the condition. When the diagnosis is confirmed, the accelerated phase should be assessed. Regarding therapy, absolute cure is achieved with an allogeneic hematopoietic stem cell transplantation (HSCT). The HSCT has better results when it is performed before the development of the accelerated phase. If indications of an accelerated phase become apparent, it is important to address hemophagocytosis and achieve remission before proceeding with HSCT. In regard to ocular symptoms, visual acuity might be improved by correcting refractive errors. Moreover, the use of sunscreen protects against skin malignancies. Early start of the rehabilitation program limits the neurologic complications, and finally, non-steroidal anti-inflammation drugs (NSAIDs) must be avoided, as they can cause bleeding events; the immunization program must be followed, and antibiotic treatment for bacterial infections must start as soon as possible [20].

### 2.5. Myelokathexis

Myelokathexis is a rare condition that causes severe chronic neutropenia and leukopenia due to the retention of neutrophils in the bone marrow. Characteristic findings include degenerative changes, hypersegmentation of mature neutrophils, and hyperplasia of bone marrow myeloid cells. Diagnosis is made with bone marrow aspiration and microscopic examination of blood samples. The affected patients’ bone marrow shows abundant neutrophil lineage cells and characteristic pyknotic nuclear lobes connected by fine chromatin filaments in the mature neutrophils. Microscopic examination of blood samples reveals >97% polymorphonucleated leucocytes. Treatment of the disease includes the administration of either G-CSF or granulocyte-macrophage-colony stimulating factor (GM-CSF) that increases the neutrophil count and reduces infection indices [21].

### 2.6. Reticular Dysgenesis

Reticular dysgenesis (RD) is a rare congenital disorder caused by mutations in the gene encoding adenylate kinase 2 (AK2). RD is defined clinically by a combination of severe combined immunodeficiency (SCID), agranulocytosis, and sensorineural deafness. Reticular dysgenesis is a rare disorder; only ~20 cases are reported. Besides the typical combination of T-B-NK-SCID and agranulocytosis, patients with RD reportedly suffer from a profound sensorineural hearing deficit. Individuals typically experience severe infections at an early stage of life, often occurring shortly after birth. Swift identification and crucial medical treatments are essential to provide a potential cure for this fetal disease. RD is presented with life-threatening infections, usually in the first days of life, accompanied by bacterial sepsis in most cases. Laboratory findings include lymphopenia with persistent agranulocytosis, T-cell numbers below the normal ranges, hemoglobin levels below the normal levels, thrombocytopenia, and bone marrow revealed hypoplasia or hypoplasia. HSCT is the only curative therapy [22].

### 2.7. Dyskeratosis Congenita

Dyskeratosis congenita is an X-linked genetic disease that is characterized by ectodermal dysplasia and hematopoietic failure. Its incidence is estimated to be 1 case per million individuals [23]. Ectodermal dysplasia of dyskeratosis congenita presents with its classic triad of cutaneous reticular hyperpigmentation, nail dystrophy, and leucoplakia of the mucous membranes. Other symptoms such as obstructed tear ducts (epiphora), developmental delay, short stature, dental caries, tooth loss, early appearance of gray hair, and hair loss may also co-exist. Hematology indices include mild neutropenia and aplastic anemia associated with high mean corpuscular volume (MCV) and elevated Fetal Hemoglobin (HbF). Infections are rarely seen [2,24].

## 3. Secondary Neutropenia with Reduced Bone Marrow Reserve

### 3.1. Drug-Induced Neutropenia

Drug-induced neutropenia is a disorder that can be caused either by decreased production or by increased destruction of neutrophils. Neutropenia caused by decreased production of neutrophils is related to chemotherapeutic drugs that can suppress the myeloid progenitor cells in the bone marrow. Increased neutrophil destruction is related to idiosyncratic drug-induced neutropenia (IDIN), where nonchemotherapy drugs are responsible for the condition. The prevalence of IDIN is 1.6–15.4 per million per year [25]. Chemotherapy drugs that cause neutropenia are alkylating agents, anthracyclines, antimetabolites, camptothecins, epipodophyllotoxins, hydroxyurea, mitomycin C, texanes, and vinblastine, while nonchemotherapy drugs such as clozapine, dapsone, hydroxychloroquine, infliximab, lamotrigine, methimazole, oxacillin, penicillin G, procainamide, propylthiouracil, quinidine/quinine, rituximab, sulfasalazine, trimethoprim/sulfamethoxazole and vancomycin are the causes for IDIN. Early diagnosis of IDIN is difficult, as patients usually are asymptomatic. A complete blood count will reveal a granulocyte count of <1500 × 10^6^/L (more often 500 × 10^6^/L), while other cell counts (red blood cells, platelets) are within normal ranges. The most important part of the treatment is the identification and cessation of the offending medication. Sometimes, due to multiple drug usage, it is difficult to determine the offensive one. After drug removal, in most cases, neutropenia will resolve, and only symptomatic treatment with antibiotics and good hygiene will be needed. The average duration for complete recovery of neutrophils is approximately 9 days. Patients with extended neutropenia may also require treatment with hematopoietic growth factors such as G-CSF [26].

### 3.2. T-Cell Large Granular Lymphocytic Leukemia

T-cell large granular lymphatic (LGL) leukemia is a proliferation of cytotoxic (CD8+) T-cell clones that cause neutropenia, anemia, and thrombocytopenia, often associated with autoimmune disorders. It affects 0.14 per million individuals [27]. Clinically, the disease is diagnosed because of recurrent bacterial infections, including cellulitis, perirectal abscesses, and respiratory infections. Other symptoms include fatigue due to anemia, increased temperature, night sweats, and decreased weight. Hepatosplenomegaly is commonly found, while some of the patients may be asymptomatic. Laboratory findings include neutropenia with absolute neutrophil count <500 × 10^6^/L. Half of the patients present with anemia and moderate thrombocytopenia. Peripheral blood smear examination reveals an increased number of granular lymphocytes with normal absolute lymphocyte number or mild lymphocytosis. These patients usually also have serological abnormalities, such as rheumatoid factor, antinuclear, antiplatelet, and antineutrophil antibodies, hyper/or hypogammaglobulinemia, positive Coombs test, monoclonal gammopathies, and increased β2-microglobulin. The diagnosis should be suspected in all patients with unexpected cytopenias and an increased number of LGLs by morphology and flow cytometry. Abnormal proliferation of CD8+ T-cells has to be shown as clonal for a definite diagnosis to be made. Polymerase chain reaction (PCR) is a widely used method with a sensitivity of 70–80%. Flow cytometry using monoclonal antibodies can also detect the clonal process of T-cell disorders [28].

### 3.3. Nutritional Deficiency

Neutropenia is caused by nutritional deficiency of vitamin B12, folic acid, and copper, and severe protein-calorie deficit in nutrition leads to multiple cytopenias rather than solely neutropenia. Patients exhibit clinical manifestations such as fatigue, decreased weight, and pale skin. Laboratory findings include anemia in complete blood count and deficiency of vitamin B12, folic acid, copper, and ferritin elements [29].

### 3.4. Viral Infections

The most common causative viruses for neutropenia include varicella, EBV, CMV, measles, hepatitis virus, and HIV. The mechanism of neutropenia caused by viral infections includes bone marrow granulopoiesis suppression, which occurs directly or through an immune-mediated process. The level of neutropenia can differ from mild to severe. Granulocyte-colony stimulating factor (G-CSF) treatment may be required in patients with severe neutropenia and detected infection [7].

## 4. Neutropenia with Normal Bone Marrow Reserve

### 4.1. Chronic Benign Neutropenia of Infancy and Childhood

Chronic autoimmune neutropenia of infancy and childhood is a common disorder, which usually resolves by the age of 3–5 years. It occurs in 1:100,000 children/year, with the mean age of onset being 7–9 months [30]. The disorder is benign despite the low ANCs. In most cases, it is detected during acute diseases, usually febrile ones. The persistence of neutropenia after the disease resolution should suspect physicians for the diagnosis. Numerous tests can be performed to confirm the diagnosis, including the identification of autoantibodies against surface antigens of neutrophils. In older children, identifying these antigens indicates further investigation for congenital immunological disorders. Screening for these disorders includes measurement of circulation T-cell receptor α/β positive, CD4+/CD8+ double negative T-cells, or serum immunoglobulin. Definite diagnosis of these conditions requires specialized immunological screening [2,31].

### 4.2. Non-Immune Chronic Benign Neutropenia

Non-immune chronic benign neutropenia more commonly appears in adults than children. Usually, it is an accidental finding in complete blood count where the degree of neutropenia is mild and is caused by an increased level of destruction of neutrophils. There are no typical clinical manifestations of the disease. Splenomegaly is seen in some rare cases of adults, usually due to increased serum concentration of pro-inflammatory cytokines and chemokines, as well as a high level of soluble cell adhesion molecules [32].

### 4.3. Benign Familial Neutropenia

Benign familial neutropenia is an autosomal-dominant inherited disease. It is usually met within specific ethnic groups, such as Americans, South African Blacks, and other African tribes. Its prevalence has been estimated to be 25–50:100 in Africans, 4.5:100 in African-Americans, 10.7:100 in Arabs, 11.8:100 in Yemenite Jews, and 15.4:100 in Black Ethiopian Jews [33]. The cause of neutropenia in this disease is unknown, and the diagnosis is usually made in the aforementioned ethnicities when other pathological causes of neutropenia have been excluded. Because of the benign course of the disease, no treatment is necessary [6].

### 4.4. Autoimmune Neutropenia

Autoimmune neutropenia is a rare disease (0.12–1.14:100 European individuals) caused by antibodies directed against neutrophil-specific antigens, leading to their destruction [34]. It includes primary and secondary autoimmune neutropenia. Anti-neutrophil antibodies, called human neutrophil allogen (HNA) antibodies, are directed against neutrophil surface glycoproteins. Diagnosis is performed directly by granulocyte immunofluorescence test, where paraformaldehyde-fixed neutrophils are incubated with serum to allow neutrophil antibodies to bind to the antigenic epitopes, and indirectly by serum granulocyte agglutination test (GAT). In this test, agglutination of neutrophils produced by IgG antibodies in the GAT is an active process, occurring in two phases: in the first phase, neutrophil reactive antibodies bind to native antigens on unfixed neutrophils, sensitizing them. In the second stage, sensitized neutrophils undergo chemostasis and move toward other polymorphonuclear neutrophils (PMNs) [35].

#### 4.4.1. Primary Autoimmune Neutropenia

Primary autoimmune neutropenia is mainly diagnosed at an early age (5–15 months), and spontaneous reduction in neutrophils occurs in almost all cases. Its incidence is 1:100,000 individuals [36]. Despite the deficient number of neutrophils, these patients rarely present serious infections. Autoantibodies are not easily detected, and the screening needs to be repeated multiple times. Autoantibodies bound to NA1 and NA2 granulocytes alloantigen type [35].

#### 4.4.2. Secondary Autoimmune Neutropenia

Secondary autoimmune neutropenia is most commonly present in adulthood. Usually, it is related to autoimmune diseases like rheumatoid arthritis, systemic lupus erythematosus, and Sjogren syndrome. It can also be related to hematological diseases, solid tumors, or immunological deficiency syndromes [35].

### 4.5. Alloimmune Neutropenia

Alloimmune neutropenia is caused as a response of the newborn’s immune system to maternal incompatible antibodies. Its incidence is 0.5–2:1000 live births [37]. Neutrophil-specific antibodies HNA-1a/1b/2a, HNA-1c, HNA-3a, and HNA-4a are identified in newborn blood. The diagnosis is usually made immediately after birth, and the resolution occurs at 2–3 months of age. The degree of neutropenia ranges from moderate to severe. Affected infants have an increased risk for skin, respiratory, and urinary tract infections, omphalitis, and fever. The treatment is usually supportive, except in septic patients, where G-CSF is indicated [38].

### 4.6. Drug-Induced Neutropenia (Antibody-Mediated)

Drug-induced neutropenia through an antibody-mediated mechanism is a rare disease with a high mortality rate. Clinical symptoms include fever, sore throat, stomatitis, pneumonia, and sepsis. Diagnosis is made by bone marrow biopsy, where bone marrow granulocytes are presented with a late maturation arrest. Treatment planning requests cessation of the offending drug. In rare advanced cases, splenectomy might be required, especially in patients with pronounced anemia and thrombocytopenia [4].

### 4.7. Infection-Related Neutropenia (Antibody-Mediated)

Neutropenia presented as a result of bacterial or viral infection is called post-infection antibody-mediated neutropenia. Diagnosis is made by a history of previous infection accompanied by laboratory results confirming the diagnosis. Bone marrow microscopy might present decreased bone marrow reserve, especially shown in patients with bacterial sepsis. Treatment is mainly aimed at treating the infection, but if bone marrow maturation is shown to decrease in microscopy, G-CSF may be required [4].

### 4.8. Hypersplenism

Patients with hypersplenism might develop mild neutropenia. Hypersplenism can be related to various conditions, such as infections, neoplasms, collagen vascular disease, hepatic diseases, and hemolytic anemia. Desolation and possible destruction of neutrophils within the spleen is the mechanism causing neutropenia. The degree of neutropenia caused by hypersplenism seems to be irrelevant to the spleen size. Diagnosis is established by imaging methods, where the spleen appears to be enlarged, along with neutropenia in laboratory results [39].

### 4.9. Maternal Hypertension

Maternal hypertension can cause neutropenia during pregnancy, which usually resolves within the first months of life. Newborns with intrauterine growth restriction, HELLP (hemolysis, elevated liver enzymes, and low platelets) syndrome, and premature rupture of membranes are at higher risk of developing neutropenia. Neutropenia, even if it is self-limited, can increase the risk of hospital infections, as it requires hospitalization of the newborn [40,41].

## 5. Diagnostic Approach to Neutropenia

Regarding the diagnostic approach of a child with neutropenia, firstly, neutropenia should be confirmed by absolute neutrophil count in peripheral blood smear [1] according to age-specific institutional reference ranges [4]. If institutional reference ranges for the pediatric population are not provided, the International Council for Standardization in Haematology (ICSH) recommends the use of published reference ranges [42]. In Table 1, we provide reference ranges for age-specific white blood cells and leukocyte differential in a routine blood count.

Apart from the absolute neutrophil count in peripheral smears, other findings that are directly related to specific diseases or disorders should be evaluated (Table 2).

Moreover, the pediatrician or laboratory physician who is called to investigate neutropenia in childhood must be familiar with the age-specific causes (Table 3).

A detailed family and clinical history should be taken. The time of onset of neutropenia, the severity of infection and the medications used must be recorded. Medications that have been associated with neutropenia are shown in Table 4.

A positive family history of neutropenia, bacterial infections early in life (e.g., infection of the umbilical cord stump), susceptibility to infections, and unexplained sudden infant death in the family should be directed to congenital neutropenia syndromes [49,50]. To date, more than 24 genes have been identified to be associated with congenital neutropenia syndromes (Table 5) [49], with the majority of published cases (60%) being associated with *ELANE* gene mutations [51]. *ELANE* gene is present in a gene cluster on chromosome 19 and is associated, apart from congenital neutropenia syndromes, with cyclic neutropenia. The protein initially produced by this elastase gene undergoes proteolytic processing to create the active form of the enzyme. Once activated, this enzyme breaks down proteins found in specialized neutrophil lysosomes called azurophil granules, as well as proteins present in the extracellular matrix. The enzyme’s activity is thought to contribute to degenerative and inflammatory diseases by breaking down collagen-IV and elastin. Moreover, this protein can degrade the outer membrane protein A (OmpA) of *E. coli* and the virulence factors of bacteria such as *Shigella*, *Salmonella*, and *Yersinia* [52]. 

The clinical physician must conduct a thorough physical examination. Growth development, mental status, and phenotypical abnormalities must be recorded, while all the systems must be examined [1]. In Table 6, we present the clinical findings observed in various disorders that can cause neutropenia.

**Figure 1:**
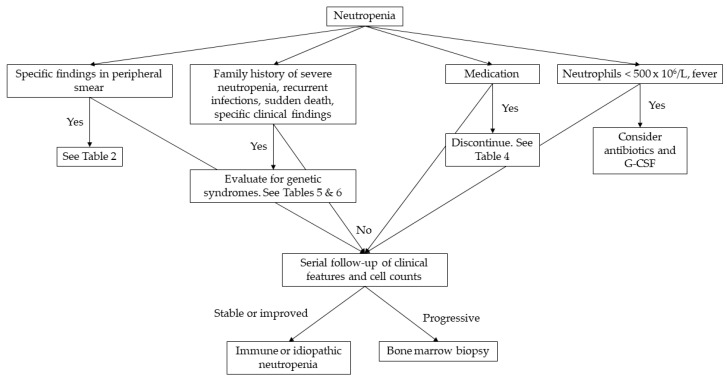
Diagnostic approach to neutropenia in childhood. G-CSF: granulocyte-colony stimulating factor.

The extent of the laboratory examinations is determined by the severity and duration of neutropenia [1]. In the pediatric population, viral infections are the main cause of neutropenia, apart from neonatal sepsis, where neutropenia is caused by bacterial infections [4]. In most cases, it is hard to distinguish whether neutropenia is the cause or the result of the infection. Nevertheless, a complete blood count should be repeated in 2–4 weeks, and if neutropenia is resolved, no further examinations are needed [1]. If neutropenia persists, complete blood count should be repeated 2–3 per week for 6 weeks to differentiate cyclic neutropenia from severe chronic neutropenia [9]. The detection of antineutrophil antibodies can diagnose chronic benign neutropenia in infancy and childhood [4]. Bone marrow aspiration must be performed when neutropenia progresses, and myelodysplastic syndrome or leukemia must be ruled out or confirmed [1,4]. In Figure 1, we provide a practical diagnostic algorithm that we believe will be helpful to any physician who will be called to manage a pediatric case of neutropenia.

## 6. Conclusions

This narrative review attempts a comprehensive and critical analysis of the current scientific data on the topic of neutropenia in children. Acquired neutropenia is usually benign and most frequently attributed to viral infections. There should be caution about drug administration in children, as this can also lead to neutropenia. Pediatricians should familiarize themselves with autoimmune disorders that can cause neutropenia. We present that clinical examination directs diagnostic investigations and clinical findings pinpointed to specific laboratory and genetic testing. Congenital neutropenia syndromes are a group of rare genetic disorders clinically manifesting with severe infections early in life. The ELANE gene should be tested in all cases of “unexplained-idiopathic” congenital neutropenia. Finally, our review concludes with a practical diagnostic approach to neutropenia in children, which can serve as a guide for the optimal handling of neutropenic patients.

## Figures and Tables

**Table 1:** White blood cells and leukocyte differential according to age (adapted from Ref. [43]).

Age	WBC	Neu *	Lymph	Mono	Eos
	Mean (95% CI)	Mean (95% CI) (%)	Mean (95% CI) (%)	Mean (%)	Mean (%)
Birth	18,100 (9000–30,000)	11,000 (6000–26,000) (61)	5500 (2000–11,000) (31)	1100 (6)	400 (2)
12 hr	22,800 (13,000–38,000)	15,500 (6000–28,000) (68)	5501 (2000–11,000) (24)	1200 (5)	500 (2)
24 hr	18,900 (9400–34,000)	11,500 (5000–21,000) (61)	5800 (2000–11,500) (31)	1100 (6)	500 (2)
1 wk	12,200 (5000–21,000)	5500 (1500–10,000) (45)	5000 (2000–17,000) (41)	1100 (9)	500 (4)
2 wk	11,400 (5000–20,000)	4500 (1000–9500) (40)	5500 (2000–17,000) (48)	1000 (9)	400 (3)
1 mo	10,800 (5000–19,500)	3800 (1000–8500) (35)	6000 (2500–16,500) (56)	700 (7)	300 (3)
6 mo	11,900 (6000–17,500)	3800 (1000–8500) (32)	7300 (4000–13,500) (61)	600 (5)	300 (3)
1 yr	11,400 (6000–17,500)	3500 (1500–8500) (31)	7000 (4000–10,500) (61)	600 (5)	300 (3)
2 yr	10,600 (6000–17,000)	3500 (1500–8500) (33)	6300 (3000–9500) (59)	500 (5)	300 (3)
4 yr	9100 (5500–15,500)	3800 (1500–8500) (42)	4500 (2000–8000) (50)	500 (5)	300 (3)
6 yr	8500 (5000–14,500)	4300 (1500–8000) (51)	3500 (1500–7000) (42)	400 (5)	200 (3)
8 yr	8300 (4500–13,500)	4400 (1500–8000) (53)	3300 (1500–6800) (39)	400 (4)	200 (2)
10 yr	8100 (4500–13,500)	4400 (1500–8500) (54)	3100 (1500–6500) (38)	400 (4)	200 (2)
16 yr	7800 (4500–13,000)	4400 (1800–8000) (57)	2800 (1200–5200) (35)	400 (5)	200 (3)
21 yr	7400 (4500–11,000)	4400 (1800–7700) (59)	2500 (1000–4800) (34)	300 (4)	200 (3)

Abbreviations: CI—confidence interval; Eos—eosinophils; hr—hour; Lymp—lymphocytes; mo—months; Mono—monocytes; Neu—neutrophils; WBC—white blood cells; wk—week; yr—year. All means and CIs are presented in × 10^6^/L. * Neutrophils include band cells of all ages and a small number of metamyelocytes and myelocytes in the first few days of life.

**Table 2:** Specific findings in peripheral smear (adapted from Refs. [1,2,44]).

Finding	Disorders
Blasts	Leukemia
Nucleated erythrocytes	Hemolytic anemia, blood loss
Hypersegmented neutrophils	Vitamin B_12_ deficiency, folic acid deficiency
Giant granules in the cytoplasm of myeloid precursors	Chediak–Higashi syndrome
Neutrophils with pycnotic nuclei	Myelokathexis

**Table 3:** Causes of neutropenia according to age of onset (adapted from Ref. [4]).

Neonate	Infant/Child	Adult
**Infection**	**Infection**	**Idiosyncratic drug reactions**
Maternal hypertension	Autoimmune neutropenia	**Infections**
Maternal antibodies	Neoplasms replacing the bone marrow	**Neoplasms replacing the bone marrow**
Constitutional neutropenia disorders	Idiosyncratic drug reactions	**Chemotherapies**
*Cyclic neutropenia*	Collagen vascular disorders	Collagen vascular disorders
*Kostmann syndrome*	Immunodeficiency disorders	Immunodeficiency disorders
*Chédiak–Higashi syndrome*	Myeloablative therapies	
	Constitutional neutropenia disorders	
	Megaloblastic anemia	
	Copper deficiency	

Common causes are in bold letters. Constitutional neutropenia disorders are presented in italic letters.

**Table 4:** Medications associated with idiosyncratic drug-induced neutropenia (adapted from Refs. [45,46,47,48]).

Medications	
Amoxicillin	Metronidazole
Benzylpenicillin	Noramidopyrine
Carbamazepine	Piperacillin/tazobactam
Carbimazole	Quetiapine
Cefepime	Salazopyrine
Cefotaxime	Sulfamethoxazole/trimethoprim
Ceftriaxone	Sulfasalazine
Ciprofloxacin	Tacrolimus
Clindamycin	Teicoplanin
Clozapine	Thiamazole
Cotrimoxazole	Ticlopidine
Ibuprofen	Tobramycin
Levetiracetam	Torsemide
Linezolid	Valganciclovir
Meropenem	Vancomycin
Metamizole (dipyrone)	Venlafaxine

**Table 5:** Known genes associated with congenital neutropenia syndromes.

Gene	Inheritance/Location	Syndromes
*ELANE* [53,54]	Dominant/19q13.3	Severe congenital neutropenia, cyclic neutropenia
*CSF3R* [55]	Dominant/1p35-p34.3	Germline mutation of CSF3R
*WAS* [56]	X-linked/Xp11.4-p11.21	Severe congenital neutropenia
*CXCR2* [57]	Recessive/2q35	Chronic neutropenia
*SBDS* [58]	Recessive/7q11.22	Shwachman–Diamond syndrome
*EFL1* [59]	Recessive/15q25.2	EFL1 syndrome
*GATA2* [60]	Dominant/3q21.3	GATA2 syndrome
*G6PC3* [61]	Recessive/17q21	Severe congenital neutropenia
*SLC37A4* [62]	Recessive/11q23.3	Glycogen storage type Ib
*TAZ* [63]	X-linked/Xq28	Barth disease
*CXCR4* [64]	Dominant/2q21	WHIM syndrome
*JAGN1* [65]	Recessive/3p25.3	Severe congenital neutropenia
*VPS13B* [66]	Recessive/8q22-q23	Cohen syndrome
*GFI1* [67]	Dominant/1p22	Severe congenital neutropenia
*HAX1* [68,69]	Recessive/1q21.3	Kostmann disease
*AP3B1* [70]	Recessive/5q14.1	Hermansky–Pudlak syndrome type 2
*LAMTOR2* [49]	Recessive/1q21	Chronic neutropenia
*USB1* [71]	Recessive/16q21	Clericuzio-type poikiloderma
*VPS45* [72]	Recessive/1q21.2	Severe congenital neutropenia
*TCIRG1* [73]	Dominant/11q13.2	Severe congenital neutropenia
*EIF2AK3* [74]	Recessive/2p11.2	EIF2AK3/Wolcott–Rallison syndrome
*CLPB* [75,76]	Recessive/11q13.4	CLPB syndrome
*STK4* [77]	Recessive/20q13	STK4 (MST1) syndrome
*SMARCD2* [78]	Recessive/17q23	SMARCD2

**Table 6:** Clinical findings were observed in various disorders that can cause neutropenia (adapted from Refs. [49,79]).

**System**	**Findings**	**Disorders**
Eyes	Congenital cataract	CLPB syndrome Charcot–Marie–Tooth
	Retinochoroidal dystrophy	Cohen disease
Heart	Arrhythmias	G6PC3 neutropenia
	Dilated cardiomyopathy	Barth disease
	Cardiomyopathy	Shwachman–Diamond syndrome
	Various cardiac abnormalities	Shwachman–Diamond syndrome WHIM syndrome (tetralogy of Fallot) G6PC3 neutropenia STK4 (MST1) deficiency
Skin	Skin xerosis eczema	Shwachman–Diamond syndrome
	Prominent superficial veins	G6PC3 neutropenia
	Poikiloderma	SCN with Clericuzio-type poikiloderma
	Partial or complete albinism	Hermansky–Pudlak type 2 AP14 defect Chédiak–Higashi disease Griscelli disease
	Fine, sparse, and light-colored hair	Cartilage hair hypoplasia GATA2
	Lymphoedema	GATA2 syndrome
	Skin angiomatosis	TCIRG1 SCN
	Petechiae (thrombocytopenia)	Shwachman–Diamond syndrome GATA2 syndrome
	Hyperpigmentation on the trunk, neck, and intertriginous areas, café au lait spots, and hypopigmented areas	Fanconi anemia
Musculoskeletal system	Weakness	G6PC3 neutropenia Axonal Charcot–Marie–Tooth disease Shwachman–Diamond syndrome
	Metaphyseal dysplasia	Shwachman–Diamond syndrome Cartilage-hair hypoplasia
	Facial dysmorphia	Cohen disease
	Palatal cleft	Shwachman–Diamond syndrome
	Hyperlaxity	Cohen disease
	Short stature and various skeletal abnormalities	Fanconi anemia
Central nervous system	Mental retardation	Kostmann disease
	Epilepsy	Shwachman–Diamond syndrome Cohen disease CLPB syndrome VPS45 syndrome
Metabolic system	Type I diabetes	Wolcott–Rallison
	Fasting intolerance and glycogenesis	Glycogen storage disease type Ib
	3-methylglutaconic acid	Barth disease CLPB syndrome
Ear	Inner ear defect	GFI1/severe chronic neutropenia GATA2 syndrome
Urogenital system	Uropathy	G6PC3 neutropenia GATA2 syndrome
	Cryptorchidism	Cohen disease G6PC3 neutropenia
	Nephromegaly	VPS45 syndrome
Findings of non-bacterial infections	HPV	WHIM syndrome GATA2 syndrome STK4 deficiency
	Mycobacterial	GATA2 syndrome WHIM syndrome

Abbreviations: HPV—human papillomavirus; SCN—severe congenital neutropenia; WHIM—warts, hypogammaglobulinemia, infections, and myelokathexis.
